# Immunotherapy biomarkers for HCC: contemporary challenges and emerging opportunities

**DOI:** 10.20517/2394-5079.2022.58

**Published:** 2022-08-29

**Authors:** Sandi Kwee, Xin Chen

**Affiliations:** 1 Cancer Biology Program, University of Hawaii Cancer Center, Honolulu, Hawaii, HI 96817, USA.; 2 The Queen’s Medical Center, Honolulu, Hawaii, HI 96817, USA.

Clinical management of advanced unresectable HCC has indelibly changed with the advent of immune checkpoint inhibitor (ICI) antibody therapy. The CheckMate-040 multi-cohort trial first demonstrated the effectiveness of an anti-PD1 antibody (nivolumab) in patients with clinically advanced HCC previously treated with sorafenib, reporting an approximately 20% overall objective response rate in such patients^[1]^. Another anti-PD1 agent, pembrolizumab, demonstrated similar response rates in its phase 2 trial^[2]^, and both agents subsequently received accelerated regulatory approval for second-line systemic treatment of HCC. The CheckMate-040 trial later showed up to a 32% objective response rate in patients treated with nivolumab plus ipilimumab (an antibody targeting CTLA-4), leading to approval of this combination regimen for second-line therapy^[3]^. With regards to first-line treatment of locally advanced or metastatic and/or unresectable HCC, the IMbrave150 phase 3 randomized trial associated the combination of bevacizumab plus atezolizumab (an anti-PD-L1 antibody) with improved overall survival over first-line sorafenib, with an objective response rate of 30% (95%CI: 25%–35%) and median duration of response of 18.1 months based on a recent extended efficacy and safety analysis of the trial^[4]^. Although remarkable for the setting of advanced HCC, these findings congruously show that most patients eligible to receive ICI therapy will not experience an objective benefit and that predictive biomarkers of treatment response will be necessary to optimize the risk-benefit ratio of immunotherapy treatment in this setting.

Notable efforts had been made to identify predictive biomarkers within the cohorts of these and other HCC immunotherapy trials. In the Keynote-040 trial, tumor PD-L1 immunohistochemical expression failed to reliably predict objective response in those treated with nivolumab plus ipilimumab or nivolumab alone^[1,3]^. The phase 1b study of atezolizumab plus bevacizumab also found PD-L1 expression on tumor and tumor-infiltrating immune cells to poorly predict progression-free survival^[5]^. Furthermore, exploratory analyses conducted using archival specimens from this and another early phase trial of atezolizumab plus bevaciumab did not associate tumor mutation burden (TMB) with treatment response or progression-free survival, although high expression of VEGF receptor 2, a T-regulatory signature, and a myeloid inflammation signature were associated with benefit from atezolizumab plus bevacizumab compared with atezolizumab alone^[6]^. In a more recent extensive biomarker analysis for the IMbrave150 trial, high expression of CD274 and intra-tumor CD8(+) cells density was associated with prolonged patient survival, while high Treg:Teff ratio and expression of HCC tumor markers, such as GPC3 and AFP, were associated with poor outcome^[7]^. Although these results require further validation, they highlight the possibility of using novel biomarkers to compensate for the lack of predictive value shown by PD-L1 expression and TMB in HCC.

Much has recently been learned regarding the impact of underlying liver diseases and the liver immune microenvironment on HCC immunotherapy outcome^[8,9]^. Meta-analysis of three phase 3 randomized trials (KEYNOTE-240, CheckMate 459, and IMBrave150) identified greater survival benefits among patients with HCC of viral etiology compared to those with non-viral HCC^[10]^. Furthermore, in a subgroup analysis of the IMBrave150 trial, greater benefits were observed among Chinese patients, a group with an extremely high prevalence of hepatitis B infection^[11]^. Recently, non-alcoholic steatohepatitis (NASH), poised to become the predominant cause of HCC in several parts of the world^[12]^, has been associated with poor tumor response to anti-PD1 antibody treatment based on rigorous experiments conducted using a NASH-associated HCC animal model and corresponding retrospective analysis of clinical data^[10]^. In addition, an updated analysis of the IMBrave150 trial reported that the overall survival benefit in the atezolizumab-bevacizumab treated sub-group with non-viral HCC was similar to those treated with sorafenib (HR: 1.05, 95%CI: 0.68–1.63), although it should be pointed out that median progression-free survival and objective response rates remained similar to the rest of the intention-to-treat population^[4]^. Altogether, such observations suggest that predictive modeling for individualizing HCC immunotherapy may need to factor in the underlying cause of liver disease or include etiology-specific biomarkers.

While the earliest investigations into mechanisms of immune escape in HCC found aberrant Wnt/beta-catenin pathway activation to be relatively common as a causative factor, additional tumor-intrinsic mechanisms contributing to poor anti-tumor immunity have been described^[13]^. Liquid biopsy platforms based on hybridization capture such as Guardant360 CDx (Guardant Health, Redwood City, CA) and FoundationOne Liquid CDx (Foundation Medicine, Cambridge, MA) hold the promise of a non-invasive strategy for broadly assessing genomic alterations potentially tied to such mechanisms. First approved as pan-cancer blood tests in the US in 2020, these platforms enable non-invasive multiplex characterization of an increasing number of cancer-associated molecular alterations, including gene mutations, amplifications, and rearrangements, in addition to potentially providing other generated biomarkers such as blood-based tumor mutation burden and microsatellite instability. Because National Comprehensive Cancer Network guidelines for HCC have, in effect, absolved clinicians from obtaining liver biopsies in patients who have a high post-imaging test probability of HCC, there are fewer opportunities to procure tissue biomarkers in HCC as compared to other cancers. This makes liquid biopsy attractive from a clinical standpoint as a means to inform on the genomics of HCC. However, with regards to predicting immunotherapy response, information from current cancer-targeted liquid biopsy panels may not suffice. It might need to be combined with other biomarkers or patient stratification to address tumor-extrinsic factors such as liver disease and the liver immune environment discussed earlier.

With regards to imaging as another non-invasive means of obtaining potential multiparametric treatment response predictors, a recent multi-omics study by Murai *et al*. that included retrospective analysis of liver MRI data from 30 patients treated with atezolizumab plus bevacizumab identified intratumoral steatosis quantified by chemical shift MRI to be associated with significantly improved progression-free survival as compared to non-steatotic HCC^[14]^. While compelling as a suggestion that MRI is a potential source of immunotherapy biomarkers for HCC, additional independent cohort studies will be needed to substantiate these results.

In summary, immune checkpoint inhibitor (ICI) regimens are the standard of care for systemic treatment of advanced, unresectable, or metastatic HCC for the foreseeable future. Because only a minority of HCC patients are expected to respond to first-line or second-line immunotherapy, biomarkers for identifying HCC tumors that are immunologically vulnerable are actively being sought. Efforts to develop biomarkers for predicting immunotherapy response in HCC have met some, although still very limited, success, while finding a reliable predictor of response has become increasingly challenging due to the growing diversity of eligible patients and immunotherapy regimens. Informing on the challenges, recent studies clearly demonstrate that the underlying liver disease and microenvironment can strongly influence HCC immune avoidance and immunotherapy response. In addition, recent studies have also revealed multiple tumor-intrinsic mechanisms of immune evasion that could behave differently depending on liver and tumor microenvironment conditions. Understanding how these factors coalesce to impact immunotherapy outcomes in advanced HCC will be crucial to finding reliable multiparametric biomarkers of immunotherapy response and to developing more effective regimens for harnessing or restoring anti-tumor immunity in HCC.
